# Household Contamination with *Salmonella enterica*^1^

**DOI:** 10.3201/eid0901.020214

**Published:** 2003-01

**Authors:** Daniel H. Rice, Dale D. Hancock, Paivi M. Roozen, Maryanne H. Szymanski, Beth C. Scheenstra, Kirsten M. Cady, Thomas E. Besser, Paul A. Chudek

**Affiliations:** * Washington State University, Pullman, Washington, USA; † Whatcom County Health and Human Services, Bellingham, Washington, USA

**Keywords:** *Salmonella enterica*, occupational exposure, household contamination, vacuum cleaner bag

## Abstract

Household contamination with *Salmonella enterica* increases when occupational exposure exists (cattle farms with known salmonellosis in cattle, a salmonella research laboratory, or a veterinary clinic experiencing an outbreak of salmonellosis). Fifteen of 55 (27.2%) vacuum cleaner bags from households with occupational exposure to *S. enterica* were positive versus 1 of 24 (4.2% without known exposure. Use of a carpet cleaner and several cleaners/disinfectants reduced, but failed to eliminate, *S. enterica* from artificially contaminated carpet.

Although most cases of nontyphoid salmonellosis in humans are foodborne, a significant number appear to be acquired from households contaminated with *Salmonella enterica* (*1*–*3*). Sources and sites of contamination include house members with clinical disease, pets with sub-clinical infection, contaminated items brought into the home, toilet bowls, carpet, floors, refrigerators, and kitchen sinks and counter tops (*1*–*6*). Culture of vacuum cleaner bag contents has been used as a tool to screen households for contamination with *S. enterica* (*1*–*3*). The purpose of the present study was to determine the frequency of contamination with *S. enterica,* as indicated by culture of vacuum cleaner bag contents, in homes in which the residents had differing levels of occupational exposure.

The contents of vacuum cleaner bags (N=79 bags), collected from household vacuum cleaners, were cultured from five groups: 1) occupants had no known exposure to livestock or *S. enterica* in the workplace (n=12), 2) one or more occupants had direct contact with livestock with no known recent salmonellosis cases (n=12), 3) one or more occupants had direct contact with cattle salmonellosis cases associated with the serovar Typhimurium (n=26), 4) occupants were exposed to cats involved in a veterinary clinic outbreak of feline salmonellosis associated with the serovar Typhimurium (n=16), and 5) one or more occupants were laboratory or field workers engaged in research on *S. enterica* (n=13).

Vacuum cleaner bags were stored at room temperature and cultured for *S. enterica* within one week of collection. The contents of each bag were cultured in duplicate. Twenty-five g of bag contents were added to 225 m buffered peptone water (BPW, Remel Inc., Lenexa, KS), and incubated overnight at 37^o^C. Preenriched samples were mixed, and 1 mL of BPW was transferred to 9 mL of tetrathionate broth (Tet, Remel Inc.), incubated overnight at 37°C, and then 100 µL of Tet was transferred to 10 mL Rappaport-Vassillladis broth (R10, Difco, Detroit, MI). The Tet tubes were incubated an additional 24 h with the R10 tubes at 37°C, and then plated onto brilliant green agar containing sulfadiazine (BGS, Difco, Detroit, MI). BGS plates were incubated for 48 h at 37°C , examined at 24 h and 48 h, and suspect colonies were biochemically screened. All *S. enterica* isolates were serotyped by the National Veterinary Services Laboratory, Ames, Iowa.

*Salmonella* organisms from all groups were found in household vacuum cleaner bags, except those from homes in which occupants had no contact with livestock or exposure to *S. enterica* (Table 1) in the workplace. *S. enterica* serovar Dublin was found in 1 of 12 (8.3%) vacuum bags collected from households with direct contact with livestock having no known recent cases of salmonellosis. Eight of 26 (31%) vacuum bags from households with occupants who had contact with *Salmonella*-infected cattle were positive. One of the positive vacuum bag samples came from a home in which an infant developed salmonellosis concurrent with an outbreak on the dairy farm where his father was employed. From households where occupants were exposed to an outbreak of feline salmonellosis, 3 of 16 (19%) of bags were positive, and from households of personnel engaged in field and laboratory based research on *S. enterica*, 4 of 13 (31%) bags were positive. All *S. enterica* isolates from households with known occupational exposure belonged to the serovar Typhimurium; as might be expected, given that all known contact exposures were with this serovar.

**Table 1:** *Salmonella enterica* culture results from the contents of household vacuum cleaner bags collected from homes with five different exposure categories

Exposure category	No. positive (%)	No. cultured	Serotypes isolated
No contact with livestock or animal salmonellosis	0 (0)	12	
Contact with livestock, no known salmonellosis	1 (8.3)	12	Dublin
Contact with livestock with salmonellosis	8 (30.8)	26	Typhimurium
Contact with veterinary clinic with many cases of cat salmonellosis	3 (18.8)	16	Typhimurium
Employment in laboratory engaged in research on *S. enterica*	4 (30.8)	13	Typhimurium
Total	16 (20.3)	79	

Since vacuum cleaners are primarily used to clean floors, the floors were likely the primary site of household contamination in this study. To ascertain the best way to remove *S. enterica* from carpeted floors (to advise affected persons), we began a study to identify a means of decontaminating carpet that was artificially contaminated with *S. enterica*. In this experiment, nine carpet segments (40 cm X 80 cm) were attached to separate sections of plywood. Each carpet segment was subdivided into four quadrants and a 15-cm X 25-cm rectangle was marked in each quadrant with indelible ink. Fresh bovine feces was injected with 10^6^ CFU/g with five serovars of *S. enterica* (Typhimurium, Dublin, Infantis, Heidelberg, and Newport), chosen for their resistance to the antibiotics ampicillin, chloramphenicol, and streptomycin. Approximately 500 g of this feces was evenly distributed onto each carpet segment by vigorous rubbing with a sponge mop. Feces-coated carpet segments were allowed to dry overnight at room temperature. Pretreatment samples were collected from the upper right and lower left quadrant of each segment by wiping a sponge (Speci-sponge, Nasco International Inc., Fort Atkinson, WI), saturated with BPW, over the surface of the carpet (10 times in one direction and 10 times perpendicular to the initial direction). The sponge was placed into a Whirl-pak bag (Nasco International Inc.) containing 25 mL BPW and mixed by using a Stomacher laboratory blender for 60 s. Serial dilutions of the BPW/carpet content suspension were spread onto MacConkey agar plates (Remel Inc.) containing ampicillin (256 µg/mL), chloramphenicol (8 µg/mL), and streptomycin (32 µg/mL) (MacACS) and incubated at 37°C overnight. Non–lactose-fermenting colonies on MacACS were counted and a subset assayed biochemically and serologically for *S. enterica*. One carpet segment was used as a control (not cleaned) and the remaining eight segments were cleaned, until free of visible soiling, with a commercial wet-vacuum carpet cleaning system along with the proprietary carpet-cleaning agent. After cleaning, two carpet segments were treated, by using the wet vacuum system, with each of; chlorhexidine (Virosan Bio-Ceutic, Boehringer Ingelheim Vetmedica Inc., St Joseph, MO, 8 oz/gal), a quaternary ammonium disinfectant (Lysol all purpose cleaner, Reckitt Benckiser Inc., Wayne, NJ, 8 oz/gal) and a phenolic disinfectant (LpH Ag, STERIS Corp., St. Louis, MO, 0.5 oz/gal). Two cleaned segments were not treated with a disinfectant. Carpet segments were allowed to dry overnight at room temperature, after which sponge samples were collected from the upper left and lower right quadrants and cultured as described.

The carpet cleaning/sanitizing experiment produced contamination levels in excess of what likely occurs naturally in carpet; however, this level was necessary to allow a measure of reduction of *S. enterica* by the selected treatments. None of the treatments was successful in eliminating *S. enterica* from carpet (Table 2). Though the differences between treatments were not significant, perhaps owing to the small sample size, carpet cleaner followed by a phenolic disinfectant resulted in the largest reduction, whereas carpet cleaner followed by chlorhexidine resulted in no observable decrease.

**Table 2:** Log_10_ CFU/mL *Salmonella enterica* (standard error) on contaminated carpet segments cleaned with a commercial carpet cleaner followed by different sanitizers

		Cleaned by a commercial carpet cleaner followed by				
	Control	No sanitizer	Chlorhexidine^a^	Phenolic disinfectant^b^	Quaternary ammonium^c^	
No. carpet segments/treatment	1	2	2	2	2	
Pretreatment	2.87	3.36 (0.18)	2.44 (0.16)	3.18 (0.02)	3.28 (0.11)	
Posttreatment	2.60	2.26 (0.09)	2.54 (0.36)	0.81 (0.81)	1.67 (0.27)	
Mean change	-0.27	-1.10 (0.27)	0.10 (0.52)	-2.37 (0.79)	-1.61 (0.38)	

^a^Virosan Bio-Ceutic, Boehringer Ingelheim Vetmedica., St Joseph, MO. ^b^LpH Ag, STERIS Corp., St. Louis, MO. ^c^Lysol, Reckitt Benckiser Inc., Wayne, NJ. ^1^ This study was performed at the Field Disease Investigation Unit, Department of Veterinary Clinical Sciences, College of Veterinary Medicine, Washington State University, Pullman, WA 99164-7060, USA.

This study confirmed the findings of others (*1*–*3*) that culture of vacuum cleaner bags is an efficient screening tool for household *S. enterica* contamination. Historically, some human salmonellosis cases have been attributed to direct contact with infected animals (*7*,*8*), while the potential for indirect contact in the home is typically not considered in public health case investigations and preventive efforts. Occupational exposure to *S. enterica* poses a potential risk to family members through inadvertent contamination of the home. When the three occupationally exposed groups were combined, Typhimurium was found in 27.2% of households. More and varied types of samples per household would likely have yielded a higher percentage of salmonella-positive homes. The vacuum bag samples in the current study were not quantitatively assayed, and some or all of the positive samples may have been contaminated at very low concentrations. However, in one household with a positive vacuum bag sample, salmonellosis developed in a family member, concurrent with this disease in cattle on the farm where another family member worked, suggesting that household exposure to *S. enterica* can be sufficient to cause an infection. The infective dose of *S. enterica*, especially for children, is not necessarily high (*9*–*11*), and circumstantial evidence exists for the acquisition of clinical infections from the household environment (*1*–*3*,*12*). For persons living in at-risk households, the risk of salmonellosis from household contamination could conceivably far outweigh the risk from food sources, and questions aimed at identifying this risk factor should be a routine part of salmonellosis case investigations. From households deemed to be at risk of environmental contamination, a vacuum cleaner bag should be collected and its contents assayed for *S. enterica*.

Carpet is a likely site of contamination in households and, once the carpet is contaminated, eliminating *S. enterica* by using conventional carpet cleaning methods is difficult, if not impossible. Use of a phenolic disinfectant resulted in the greatest reduction of *S. enterica* in carpet; however, this product may not be suitable for use in carpet due to the possibility of hazardous residues. Previous studies have reported the persistence of salmonellae when various disinfectants and cleaning strategies are used (*4*,*13*). The rapid buildup of bacteria in carpet under normal usage, the subsequent difficulty removing bacteria from carpet during cleaning, and the ability of bacteria to survive in carpet and other fabrics for many months has been documented (*14*,*15*).

The current study indicates that precautions are warranted for the home environments of personnel who regularly have contact with livestock or who have occupational exposure to *S. enterica.* Preventive measures such as having noncarpeted entry areas and removing footwear before entering living areas should be taken to minimize the chances of contaminating the home environment, especially when households have members who are predisposed to infection with enteropathogens by factors such as age or immunocompromised status.
